# Health information systems in developing countries: case of African countries

**DOI:** 10.1186/s12911-021-01597-5

**Published:** 2021-08-04

**Authors:** Aimé Patrice Koumamba, Ulrick Jolhy Bisvigou, Edgard Brice Ngoungou, Gayo Diallo

**Affiliations:** 1 Bordeaux Population Health (BPH), UMR1219, Applied Health Informatics Research Team (ERIAS), Univ. Bordeaux, 33000 Bordeaux, France; 2 Research Unit in Epidemiology of Chronic Diseases and Environmental Health (UREMCSE), University of Health Sciences, BP 11587, Libreville, Gabon; 3 UMR 1094 Inserm parter IRD-Tropical Neuroepidemiology (NET), Faculty of Medicine of the University of Limoges, 87025 Limoges, France

**Keywords:** Health information systems, Governance, Health data, Developing countries, Performance

## Abstract

**Background:**

In developing countries, health information system (HIS) is experiencing more and more difficulties to produce quality data. The lack of reliable health related information makes it difficult to develop effective health policies. In order to understand the organization of HIS in African countries, we undertook a literature review.

**Methods:**

Our study was conducted using the PubMed and Scopus bibliographic search engines. The inclusion criteria were: (i) all articles published between 2005 and 2019, (ii) articles including in their title the keywords "health", "information", "systems", "system", "africa", "developing countries", "santé", "pays en développement", "Afrique", (iii) articles that are written in English or French, (iv) which deals with organizational and technical issues about HIS in African countries.

**Results:**

Fourteen retrieved articles out of 2492 were included in the study, of which 13 (92.9%) were qualitative. All of them dealt with issues related to HIS in 12 African countries. All 12 countries (100.0%) had opted for a data warehouse approach to improve their HIS. This approach, supported by the DHIS2 system, has enabled providing reliable data. However, 11 out of the 12 countries (92.0%) frameworks were aligned with funding donors’ strategies and lacked any national strategy.

**Conclusion:**

This study suggests that the lack of a national health information management strategy will always be a threat to HIS performance in African countries. Ideally, rigorous upstream thinking to strengthen HIS governance should be undertaken by defining and proposing a coherent conceptual framework to analyze and guide the development and integration of digital applications into HIS over the long term.

## Background

The performance of a health system is strongly related to the quality of produced health information. Quality health information (credible, coherent, up to date, etc.) allows the health system to be effective and efficient in terms of decision-making by the health managers in terms of resource planning, monitoring and evaluation of actions undertaken for health, epidemiological surveillance [1]. It is on the basis for example, of quality health information that decision-makers not only have visibility on the performance of their health system, but also have the capacity to react more rapidly to health needs.

Every year, for example, the World Health Organization (WHO) publishes figures on the world’s health situation in order to measure member countries’ health outcomes. That shows the importance of well-structured Health Information Systems (HIS). The latter properly produces, analyses, stores and shares reliable and accurate information for decision support at all levels of the healthcare system pyramid. Consequently, investing in the implementation of HIS has become a great challenge for health care systems in developing countries. Faced with these major health issues, coupled with the achievement of Sustainable Development Goals (SDGs), and focusing on its third goal, related to healthcare entitled "Healthcare and well-being", a highly-performed health information system could play a major role to enhance monitoring of indicators.

### Relationship between a health system and a health information system

To better establish the relationship between a health system and a health information system, it was necessary for us to first of all understand what a health system is, just as defined by the WHO. According to the WHO’s definition, a health system is defined as "all organizations, individuals and actions whose primary purpose is to promote, restore or maintain health" [2]. To achieve this objective, the health system is founded on a conceptual framework with six pillars outlined as follows.

- **Leadership and governance**: involves the definition of national health policies, roles and responsibilities of all the stakeholders;
- **Health finance**: implies having the necessary funds to finance expenditures related to the health needs of the people thereby enabling them to benefit from services that effectively deliver needed services;
- **Health staff**: implies that the health system must have a sufficient and qualified human resource in the healthcare domain, in order to sustain its performance;
- **Essential medication and technologies**: implies a more efficient health system, which requires proper availability of medication and technologies;
- **Service delivery**: implies an organization of health service delivery that efficiently corresponds and responds to the spatio-temporal health needs of populations;
- **Health information**: implies ensuring the availability of health information necessary for decision making. The necessity of reliable and accurate information in order to plan, monitor and evaluate undertaken activities or to take action so as to achieve the objectives as defined in health policies.

The conceptual framework defined by the WHO clearly demonstrates that the organization of any health system must be perceived in a systemic approach by taking into consideration all its different stakeholders, including those related to its information system. It is also defined by the WHO as "an organized system that integrates the collection, processing, communication and use of data necessary to improve the effectiveness and efficiency of health services through better management at all of their levels". The latter definition highlights the transversal nature of a HIS, in that it integrates the entire structural dimension of a health organization.

Looking at the HIS topic from the above approach, it is clear that no health system aspiring to be effective can become effective without having a HIS capable of collecting, processing, analyzing and making available reliable and timely information for the purpose of planning, monitoring and evaluating health actions.

### Problem statement

Although having a HIS is a necessity for any health systems, many developing countries still have nowadays difficulties in obtaining reliable and accurate health related information. Due to that issue, they are unable to obtain good health results. Consequently, their performance is far from meeting the needs of the population [2].

This literature review aims to provide an overview of the factors that influence the optimal functioning of HIS in limited settings and resources countries.

## Methods of operation

The study was carried out from February to July 2019 in three stages: (i) relevant articles research, (ii) articles selection, performed by carrying out a complete analysis of each article according to the publication dates and (iii) proceeding to the global analysis of all articles in a general synthesis.

### Research strategy

For articles look-up, we used the Scopus and PubMed databases and their research systems to conduct our bibliographic search. To do so, we searched for relevant articles dealing with national HIS performance (performance is understood here as organizational, technical and behavioral determinants). In the bibliographic research system of these databases, we used the following keywords: "health", "information", "systems", "system", "africa", "developing countries", "Africa".

Using the filters provided by the search engines (for example the publication date), relevant articles were selected on the basis of the criteria described in Table 1.

**Table 1:** Inclusion criteria

*Inclusion criteria*
Original articles written in English or French, peer-reviewed, published between 2005 and 2019 Research articles which addresses the performance factors of national HIS in developing countries

To lookup the databases, we exclusively relied on the logical operator "OR", because the use of the logical operator "AND" or the combination of both logical operators "OR" and "AND" enabled us to obtain a reduced number of articles, which were unfortunately not relevant for our study. As a matter of fact, we found a limited set of articles dealing with information systems issues related to specific diseases, such as malaria or cancer. As these articles do not comply with the initial objective of our study, which was to examine articles dealing with the HIS at large, in a systemic approach.

**Fig. 1:**
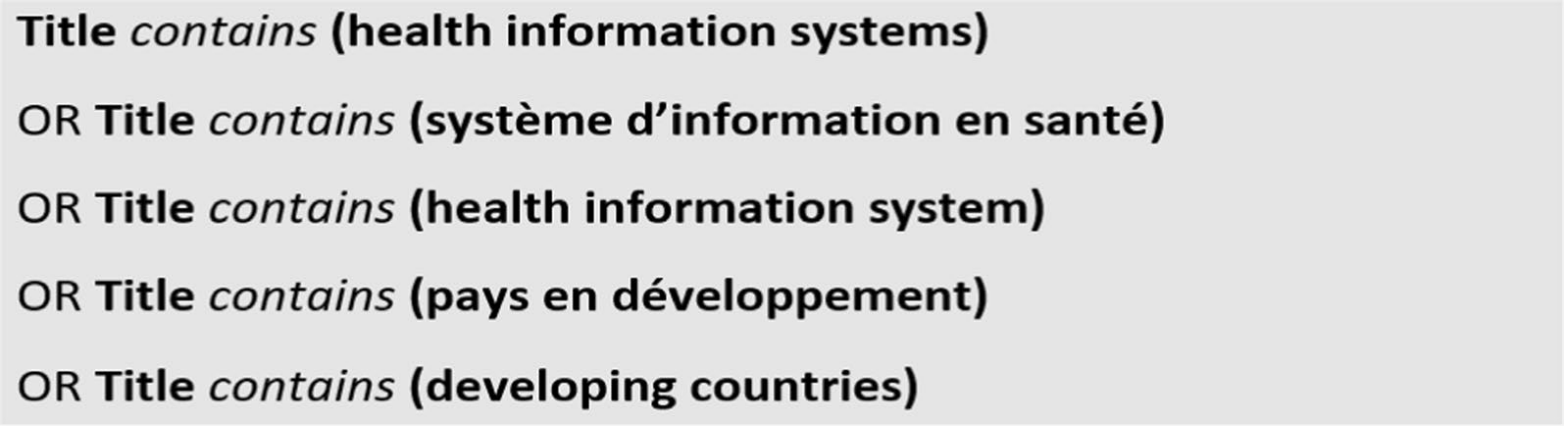
Bibliographic research equation on the Scopus database

**Fig. 2:**
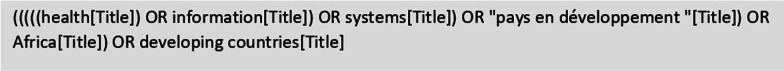
Bibliographic search equation on the PubMed database

By choosing to use only the logical operator "OR" in our bibliographic search equation, our aim was to obtain the maximum number of articles and later on select the most relevant one. This strategy allowed to obtain few relevant articles that perfectly met the objective of the study, despite some false positives (many articles not related to the study topic). The two search algorithm that allowed us to select our articles is depicted respectively in Figs. 1 and 2.

### Selection of relevant articles

All articles meeting the above inclusion criteria were eligible for inclusion in the study. Firstly, on the basis of these criteria, we carefully reviewed each article title in order to reject articles whose title, although incorporating some of the defined keywords, did not correspond to the objective of the literature review. In other words, not all the articles that focused on information systems specific to certain health issues rather than the problems of national HIS were eligible for our review.

Secondly, we used the Performance of Routine Information System Management (PRISM) approach from the United States Agency for International Development (USAID) to review the 14 articles selected for the study. The choice of PRISM was motivated, on one hand, by the fact that all reviewers had been previously trained in this approach, on the other hand, it makes it possible to evaluate the performance of a HIS on the basis of each of its three determinants, i.e., technical, organizational and behavioral for the analysis of the 14 articles selected in our study, we opted to examine only the organizational and technical determinants. The behavioral determinants were not examined, as they required the administration of a questionnaire to the various actors involved in the HIS. This was not adapted to our study, whose approach was to review the literature.

The selected articles were examined using a grid validated by all the reviewers. On that grid, the two selected determinants for the study were evaluated on the specific aspects dealt with these articles, namely HIS governance (HIS legal framework, national HIS strategies, etc.), data quality (data completeness, data reliability, data relevance, data accuracy, data timeliness, etc.), information technology (IT), data warehouses, HIS evaluation and DHIS digital solution (Fig. 5).

The analysis of these aspects related to the organizational and technical context of HIS, allowed to identify the strengths and weaknesses regarding the implementation of HIS in developing countries at the organizational and technical level (Table 2).

**Table 2:** Some strengths and weaknesses of HIS in developing countries

Organizational issue	Technical issue
*Strengths* Definition and validation of the data to be collected by all stakeholders Review and harmonization of data collection tools to avoid data overlap and duplication Definition of harmonized national indicators Validation of a national data warehouse, sometimes cohabiting with existing subsystems Consensual choice of essential indicators by theme Creation of a HIS coordination unit housed at the level of the Ministry of Health in some cases *Weaknesses* HIS not sufficiently taken into account in national health policies, making it difficult to ensure the sustainability of HIS at the end of projects Inadequate institutional management of HIS Coherent conceptual frameworks insufficiently defined Dependency on external donors for HIS funding	*Strengths* Possibility to contextualize and adapt DHIS to nationally validated models Consensus approach to a system Removal of redundant data and improvement of data quality at the central level Possibility to enter DHIS information from a mobile phone or a paper data collection medium Storage and centralization of data in a single database *Weaknesses* Design (of DHIS) taking insufficient account of the needs of data-producing structures because the collection and transmission of aggregated data more helps decision-making at the central level and not the local one Unable to collect primary data in DHIS (individual patient data) DHIS does not allow the linking of care data with those of other systems such as health insurance

## Results

**Fig. 3:**
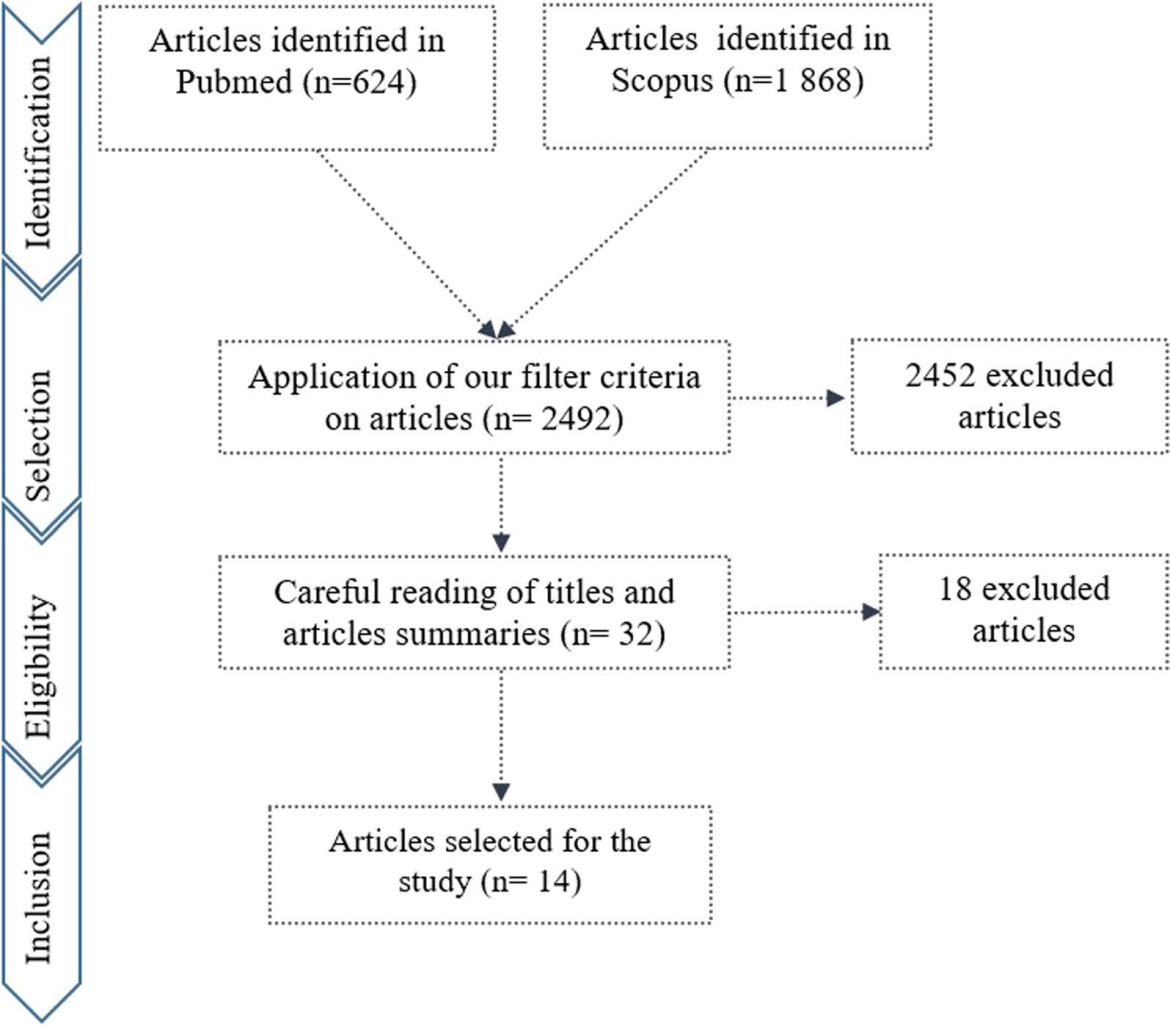
PRISMA process diagram

Based on the keywords selected to constitute the basis of our bibliographic research, we identified a total of 2492 articles: 624 identified in PubMed and 1868 identified in Scopus. Among these articles, 2460 articles dealing with information systems specific only to certain health issues were excluded from the study. Thirty-two (32) articles were relevant according to the selection criteria (full articles, written in English or French, peer reviewed and published between 2005 and 2019). Then, we carefully reviewed the titles and abstracts of the 32 articles to check whether the technical and organizational aspects of the HIS on which our articles should be examined were taken into consideration. This step, selected 14 articles corresponding to the objectives of our study (Fig. 3).

Faced with public health challenges, such as SDGs (e.g., by 2030, to reduce the global maternal mortality ratio to less than 70 per 100,000 live births), the essential role of information in a health system is increasingly highlighted in all target countries [3]. Indeed, reliable and solid health information facilitates, on one hand better monitoring of SDGs indicators and on the other hand, the development of health policies based on established facts. Nowadays, unfortunately, due to a lack of reliable data (i.e., updated, consistent, etc.), HIS in many countries are struggling to meet information needs in order to monitor their health indicators, such as the main reasons for hospital visits or the main causes of death [4].

**Fig. 4:**
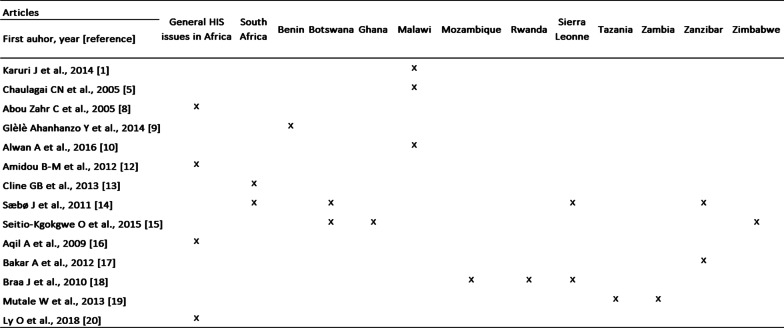
Places concerned by the information systems studied in our study

**Fig. 5:**
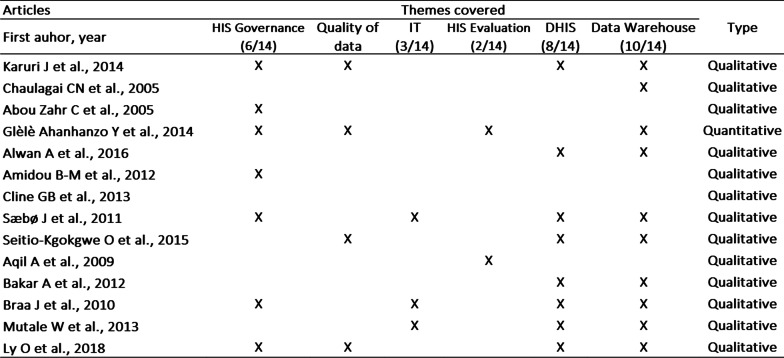
Themes covered in the 14 articles examined

A systematic review of the 14 resulted scientific articles enabled us to understand the state of health information management in some African countries in which such information had been subjected to peer reviewed scientific publications (Fig. 4). These selected articles dealt with issues related to the evaluation, organization, data quality, improvement or development of integrated information systems or data warehouse (Fig. 5).

Four articles which focus on general HIS issues in Africa [8, 12, 16, 20] and 10 others dealing with HIS-specific issues from 12 countries were identified: South Africa [13, 14], Benin [9], Botswana [14, 15], Ghana [15], Malawi [1, 5, 10], Mozambique [19], Rwanda [19], Sierra Leone [14, 18], Tanzania [19], Zambia [19], Zanzibar [14, 17], Zimbabwe [15]. Although the literature on HIS in these countries has been limited, the analysis of the available literature enabled to identify the organizational and technical structure of each of these HIS.

From the 14 selected articles, 13 (92.9%) were qualitative in nature. All 10 articles dealing with HIS issues from 12 African countries address the topic of health data warehouses, 10/10 (100%).

### Insufficient integration of stakeholders and coordination in the implementation of the HIS

A health system, insofar as it involves several actors to achieve its objectives, should also proceed by involving all these actors in the implementation of a reliable HIS strategy.

The review of the literature shows that taking into account the needs of all stakeholders in the health system is the common point between the countries identified in the study to set up HIS in Africa, although these systems have been implemented in different ways.

The structuring of health systems can be seen as follows:

- the sub-system that we can call the public sector which manages all the activities of the health structures that belong to the government;
- the sub-system that we can call the private sector which manages all the activities of health structures owned by individuals who, in their personal capacity and under their personal responsibilities, carry out activities of general interest in the health field;
- the sub-system that could be referred as the para-public sector, which manages all the activities of health structures for which the government and private individuals are all managers at the same time. For example, health care facilities owned by private individuals or companies, but receiving subsidies from the state and the assignment of staff paid by the state funding.

It is by drawing on their national health plans and involving all stakeholders, such as these different health sectors (public, para-public and private), health insurance companies, etc., that some countries have identified the needs and defined all the aspects to be taken into account in the conceptual phase of their HIS [5]. Although the development of national HIS strategies has not been sufficiently highlighted, it has nevertheless been noted in the literature that some countries that have taken into account the needs of all stakeholders have put in place coherent strategies for the integrated management of their HIS [6]. However, it should be noted that these strategies are generally short termed in nature, often after the project is implemented [7]. Insufficiently asserted coordination supported with well-defined roles and responsibilities also makes it more difficult to ensure the sustainability of these integrated HIS, making them less and less effective with doubtful information quality. This situation tends to promote the fragmentation of HIS within actors using specific information systems to collect the specific data they need. This fragmentation of the HIS is sometimes encouraged by some funding organizations that prioritize certain subsystem needs to the detriment of others and the global needs of the integrated HIS [8]. Health programs collecting more specific data to meet the objectives of their donor agencies have little involvement in more comprehensive strategies for developing and managing more integrated HIS [9]. This explains the fact that many HIS projects have experienced governance problems as a whole [10]. This is why some countries such as Malawi have optimized the quality of their data by asserting their leadership, including by implementing a national strategy. It has also been observed that 11 of the 12 countries under study (92%) did not have a national strategy, but rather strategies implemented under a specific project. Malawi is the only one that has a national strategy to which all actors, including donors, must align. This strategy was accompanied by a procedural guide to facilitate the design of a HIS integrating all sub-systems [11].

Despite the implementation of coherent strategies such as national health plans that integrate the development and management of HIS, it is clear that insufficient integration of all stakeholders and coordination of HIS is not conducive to the production of comprehensive data. Indeed, collecting data from multiple non-harmonized tools makes it difficult for managers to fill out various paper or electronic forms at the same time for several programs [12]. As a consequence of the inadequacy and reliability of data in regular HIS, some countries are forced to conduct ad hoc survey based on already validated data collection methodologies in order to have health information for decision support such as Service Availability Readiness Assessment survey, demographic and health survey, etc. to estimate certain necessary indicators [13].

In 2007, the WHO, like many other partners working to improve HIS established some mechanisms to align their support with national strategies, policies and procedures developed by partner countries [14]. In this perspective, some countries have undertaken to assess and develop their national HIS management policies. The fact that all the initiatives should be aligned to these agendas and policies for the identification and harmonization of indicators is one of the first requirements of this approach [15, 16].

### Organization of technical and infrastructural support for the implementation of HIS

To enable the considered countries to achieve better health outcomes, several funding agencies had advocated the integration of data into Data Warehouses. To materialize this recommendation requires the commitment of all actors to the plans and strategies implemented by the countries. In this regard, some mechanisms to align international efforts with national systems have been set up, including the International Health Partnership (IHP+) and the Partnership for Statistics for Development in the 21st Century (PARIS21). Some initiatives, in line with this logic, have been undertaken with the District Health Information System (DHIS) software, among others, in South Africa, Sierra Leone, Zanzibar and Botswana [14].

In South Africa, the approach had been to validate a standard model on which to build a data warehouse (DW) based on the DHIS software. This DW operates in parallel with other existing subsystems. It is notified at all levels of the health pyramid and subsystems, regardless of their specific needs. At the level of three other countries, the approach was to integrate all subsystems into a single DW. In Botswana for instance, it was intended to integrate all the paper forms of the subsystems into the DHIS software without any prior standardization, resulting in data overlap and duplication. Zanzibar and Sierra Leone, for their part, opted for a consensual (with all stakeholders) sorting of data from the different sources before their integration into a unique DW. Although using the same approach in standardizing data to be pre-integrated, their recording into the data warehouses was different in the two countries. In Zanzibar, paper-based forms were used to collect data from health facilities and made available to health districts, which were then filled in the DW. To overcome the errors which may occur with a manual process associated with the use of paper forms, the Zanzibar Ministry of Health had undertaken a project to use the OpenMRS clinical management software at the health facility level for processing and recording data in the DHIS warehouse. However, this OpenMRS-based project is experiencing difficulties in its implementation due to limited local technical capacity [17].

Sierra Leone has had an approach that takes into account the context of unequal distribution of technical infrastructures throughout the country. The adapted and validated digital DHIS solution served as a repository for the national HIS at all levels of the health system. The adaptation of DHIS was done in such a way that in areas with a digital infrastructure, this repository exchanged data with a hospital management software (HMS), such as OpenMRS where the SDX-HD standard facilitates this data exchange. The DHIS solution also provides the ability to enter data from paper or mobile phones. It is therefore a model that allows data to be recorded at all levels and aggregated in one place [18]. This evolutionary approach, based on a collaborative architecture, has enabled Sierra Leone health system to have increasingly accurate health data that is accepted by several organizations that use it.

Six other countries (Ghana, Mozambique, Rwanda, Tanzania, Zambia and Mozambique) which received support from the International Health Partnership have used the DHIS software to implement their HIS. In Zambia, Rwanda and Tanzania for instance, the software has been adapted by standardizing and computerizing the various registers to collect data on care delivery from the health facilities. While Ghana and Mozambique have adapted the software from the standardized tools collected. This, in order to record aggregated data at the facilities, district and regional levels [19]. For the majority of cases, initiatives to implement integrated HIS in the different targeted developing countries have been technically supported by the use of the DHIS solution. This digital solution, with open source code, has enabled several projects, even with various approaches, to digitize data management and analysis from the health district to the central level [20]. At the structural level, DHIS was either filled in from the paper-based supports which allowed the collection of care services, or by using an Extract, Transform & Load (ETL) process that extracts data from the given HIS to transform them and load them into a central DW.

### Presentation of the common platform used in the HIS projects in Africa: DHIS 2

DHIS 2 is an open source software platform for the integration, analysis and dissemination of routine health data developed by the Health Information Systems Program (HISP) with the support of the Department of Computer Science at the University of Oslo in Norway. Initially designed and developed for data collection at the level of basic health committees and community information systems in health districts, the platform evolved into a web-based version in 2006 with an adaptation at the national level, hence DHIS2 for version 2. It is oriented towards the capture of aggregate data from health programs. The application has a specific module, the "tracker" that can be configured to allow data to be recorded in the most granular way possible and facilitate automated compilation. This module, which is far from playing the role of an electronic patient record, is more oriented towards data specific to certain health programs and almost not towards care services with more complex data.

## Discussion

### Main lessons learned

The implementation of an HIS must take into account the process of data collection, analysis processing and transmission at all levels of the health pyramid. It must support decision-making in health planning, epidemiological monitoring and evaluation of health actions. This requires that the development of HIS be supported by sufficient governance with policies integrating HIS-related aspects, including harmonized indicators, forms (electronic or paper-based) for integrated data collection, and databases that are easy to use and accepted by users.

From the conducted literature review, it mainly emerges that governance, a crucial element for an efficient HIS, has not been sufficiently taken into account in most of the projects to set HIS upin Developing Countries. The weak involvement of all stakeholders, together with weak leadership expressed by States and insufficient coordination of HIS actors are among the main factors that have often threatened the sustainability of these HIS [10]. This is clearly highlighted by the results of some HIS evaluations following the WHO Health Metrics Network (HMN) framework and the US Agency for International Development (USAID) Performance of Routine Information System Management (PRISM) tool [9, 10]. It appears that for most cases, these HIS implementation projects are supported by external donors, with an involvement limited to a simple consensus between actors without a regulatory coordination framework at the country level [19]. Although stakeholders have often been involved in the phase of identifying information needs, harmonizing collection tools, defining data to be collected and indicators, it is unfortunately worth noting that the absence of a framework that is enforceable against all does not always guarantee establishing an integrated HIS in the long term [18]. The development of policies and strategies is an asset for the continuation of an integrated HIS after the withdrawal of the external donors, since the implementation of these HIS is based more on the policies of donor agencies often tailored to their own needs over national needs.

Some countries that have set HIS projects up on the basis of national strategies have sometimes had better data [17]. However, overall, it can be observed that despite these few successes, a number of weaknesses persist, including those related to inadequate policies and insufficient data centralization, resulting in often incomplete data. To maintain this momentum for improving data quality, it is appropriate for the ministries in charge of health to assert their leadership by developing national information management policies and strategies by defining the roles and responsibilities of the various actors with coordination structures.

The use of information and communication technologies as a support for the HIS to collect, process and disseminate health information is widely observed in DC. The implementation of the context-specific DHIS solution makes it easier than ever to collect and transmit information to central level to support decision-making [20]. However, it is unfortunate that the non-systemic structure of this IT (Information technology) solution, which takes into account very little (if any) the health related data at the structure level, undermines the latter category of actors (local structures) who, in addition to producing the data, are their first users.

### Limitations of the study

Our review, in addition to being less exhaustive in terms of representativeness of the countries whose HIS were analyzed in this study (12 out of 54 African countries), has a low number of articles retained. In addition, out of the 12 countries whose HIS issue were analyzed, 11 were English-speaking countries. However, the reality of HIS management in English-speaking countries may not be the same as in French-speaking countries, even if the health problem remains similar. It is therefore relevant to deepen such an initiative at the level of French-speaking countries and establish differences if any.

## Conclusion

The implementation of an efficient and effective information system implies in its design a systemic approach. This approach takes into account the global consideration of the needs of stakeholders at all levels of decision-making, including at the level of the health care structure. Although not sufficiently implemented in practice, because it is done in a consensual way without an enforceable framework with coordination, it has been observed that most projects initiated in Africa to set up or improve HIS have integrated this systemic approach by taking into account the needs of various actors. The main concern would be that this systemic approach is often not supported by strong leadership from department in charge of the health sector. Nevertheless, during the time of a project with a consensus between stakeholders, some countries have managed to obtain quality health data. This leaves an optimism that with a coherent and unified governance in terms of the management of state health information, better health indicators can be achieved. In such a case, IT solutions such as DHIS alone are not enough. It requires that during the development of any HIS, rigorous upstream reflections on strengthening its governance should be carried out by defining and proposing a coherent conceptual framework. The latter will make it possible to analyze and guide the development and integration of IT solutions into the HIS.

## Data Availability

Not applicable.

## Abbreviations

WHO: World Health Organization
HIS: Health information systems
HMS: Hospital management software
NHIS: National Health Information System
IT: Information technology
SDGs: Sustainable development objectives
DW: Data warehouse
DHIS: District Health Information System
USAID: US Agency for International Development
HMN: Health Metrics Network
PRISM: Performance of Routine Information System Management
IHP+: International Health Partnership
HISP: Health Information Systems Program
SOGIM: Société Gabonaise d’Informatique Médicale
